# Acceptance and Tinnitus Handicap in Chronic Tinnitus: Associations With Sleep Quality and Depression—A Cross‐Sectional Study

**DOI:** 10.1002/brb3.71078

**Published:** 2025-11-19

**Authors:** Sevgi Kutlu, Zehra Aydogan, Kübra Binay Bolat, Nazife Öztürk Özdeş

**Affiliations:** ^1^ Department of Audiology Faculty of Health Sciences, Ankara University Ankara Türkiye; ^2^ Department of Audiology and Speech Disorders, Institute of Health Sciences Ankara University Ankara Türkiye

**Keywords:** depression, sleep quality, tinnitus, tinnitus acceptance, tinnitus handicap

## Abstract

**Purpose:**

This study aims to examine the association of tinnitus acceptance on sleep quality and depression in chronic tinnitus patients, addressing a gap in the literature on acceptance processes and quality of life.

**Methods:**

A total of 130 patients (47 female, 83 male; mean age 46.75 ± 14.02) were assessed using the Tinnitus Acceptance Questionnaire (TAQ), Tinnitus Handicap Inventory (THI), Beck Depression Inventory (BDI), and Pittsburgh Sleep Quality Index (PSQI). Correlation, linear regression, and logistic regression analyses were performed. Acceptance scores were divided into “low” and “high” groups (median 41.0).

**Results:**

Tinnitus acceptance was significantly associated with depression and sleep quality. A strong negative correlation was found with THI (*r* = –0.667, *p* < 0.001), and moderate negative correlations with BDI (*r* = –0.438) and PSQI (*r* = –0.401). Regression analyses identified THI as the only significant predictor of tinnitus acceptance (*β* = –0.047, OR = 0.95), while BDI and PSQI were not independent predictors. Partial correlation and multicollinearity tests confirmed that these associations remained significant after controlling for shared variance. This indicates that the relationships were not only attributable to overlapping item content.

**Conclusions:**

Higher levels of tinnitus acceptance were associated with lower tinnitus distress, fewer depressive symptoms, and better sleep quality. These relationships are correlational and should be interpreted with caution. Integrating acceptance‐based components into psychological support programs may be a promising approach, which warrants further confirmation in longitudinal and interventional studies.

## Graphical abstract

This study examines the associations between tinnitus acceptance, sleep quality, and depression in individuals with chronic tinnitus. A total of 130 participants (mean age = 46.8) were assessed using the TAQ, THI, BDI, and PSQI. Tinnitus acceptance showed a strong negative correlation with THI and moderate negative correlations with BDI and PSQI. Regression analyses identified THI as the strongest predictor of tinnitus acceptance, while depression and sleep quality were not independent predictors. Partial correlation and multicollinearity tests confirmed that these relationships persisted after accounting for shared variance. Higher acceptance was associated with lower tinnitus distress, fewer depressive symptoms, and better sleep quality. These findings highlight correlational links between tinnitus acceptance and psychological well‐being, suggesting that acceptance‐based approaches may contribute to improving quality of life in tinnitus patients.

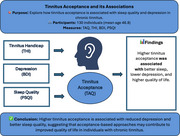

## 1 Introduction

Subjective tinnitus is defined as the subjective perception of sound in the ears and/or head in the absence of an external acoustic stimulus (Hallam et al. 1984). It has been reported that 10% of young adults, 14% of middle‐aged adults and 24% of older adults suffer from various types of tinnitus (Jarach et al. 2022).

Chronic tinnitus has been found to be associated with changes in brain structures in the auditory and limbic systems. High rates of psychiatric comorbidity have been reported in this group, particularly in relation to changes in limbic structures (Besteher et al. 2019). In addition to the occurrence of neurological alterations, chronic tinnitus is also frequently associated with a range of systemic comorbidities, including cardiovascular diseases, metabolic disorders, gastroesophageal reflux disease, autoimmune conditions, pulmonary diseases, and allergic rhinitis, reflecting its complex and multifactorial nature (Maihoub et al. 2025).

The negative impact of tinnitus on psychological well‐being has been demonstrated in many studies. Psychological symptoms such as anxiety, depression, high stress and mood disorders have been reported due to tinnitus (Langguth et al. 2013; McCormack et al. 2016). Tinnitus therapy aims to reduce the severity of tinnitus and minimise its impact on daily life, focusing on strategies to improve the patient's quality of life. Several psychological techniques have been developed to reduce the negative effects of tinnitus. Cognitive behavioural therapy is aimed at managing tinnitus and related problems (Andersson 2002). Within this context, psychological acceptance has emerged as a key mechanism of change, particularly in third‐wave behavioural therapies such as Acceptance and Commitment Therapy (Hayes et al. 2006). Psychological acceptance is the recognition and experience of current thoughts, feelings, memories and bodily sensations without trying to change or control them by accepting a situation as it is (Hayes and Pierson 2005). Acceptance refers to a situation in which tinnitus and related thoughts and feelings lose their functional impact on the individual's overt behaviour (Moschen et al. 2019). Psychological factors such as tinnitus acceptance have recently gained more ground in tinnitus research and are one of the main targets of psychotherapeutic treatments (Weise et al. 2013). It has been emphasised that tinnitus acceptance may be a crucial factor in explaining the heterogeneity of tinnitus severity (Hesser et al. 2015). Acceptance of tinnitus is considered an important element of therapeutic change in behavioural treatment approaches and professional counselling for tinnitus (Moschen et al. 2019). Research emphasises that the perception of tinnitus is largely shaped by psychological elements such as thoughts, emotions, personality traits and coping strategies (Durai and Searchfield 2016; Ooms et al. 2012). Research has shown that assessing acceptance processes in people with tinnitus is a useful measure in experimental investigations of psychological treatments for tinnitus, and that acceptance of tinnitus is associated with better functioning and well‐being (Hesser et al. 2015; Westin et al. 2008).

Although the negative effects of tinnitus on individuals' quality of life and psychological approaches aimed at mitigating these effects have been studied for a long time, information on the relationship between tinnitus acceptance and sleep quality and depression is rather limited. For this reason, the focus of the current study is on tinnitus acceptance and the main aim of the study is to investigate the association between tinnitus acceptance, sleep quality and depression in individuals with chronic tinnitus. Our study aims to provide a valuable guide for clinical practice and contribute to individualised treatment methods by identifying the focus of psychotherapeutic interventions. The results will be a valuable resource for both researchers and clinicians by highlighting the importance of tinnitus coping strategies and acceptance‐based approaches.

## 2 Material and Methods

The present study was conducted in accordance with the principles of the Declaration of Helsinki. The ethical approval of the study was obtained by the Ankara University Faculty of Medicine Human Research Ethics Committee (decision number: İ02‐108‐25). The study was conducted in the Ankara University Faculty of Medicine İbni Sina Hospital Ear Nose and Throat Department, Audiology, Balance, Speech Disorders Diagnosis and Rehabilitation Unit. Participants were consecutively selected from among patients who attended the audiology outpatient clinic with complaints of tinnitus between February 2025 and April 2025.Those who volunteered to participate in the study received a consent form and information about the study and anonymisation standards. To determine the sample size, a statistical power analysis was conducted using the G*Power program. The sample size was determined as 120, assuming, a power of 95%, and an α‐type error estimate of 0.05. The present study included a total of 130 participants, providing sufficient power to detect the expected effect.

### 2.1 Participants and Procedure

The present study comprised a total of 130 participants, 47 female and 83 male, with a mean age of 46.75 ± 14.02 years, suffering from chronic tinnitus. Inclusion criteria were as follows: 18 years of age or older, subjective tinnitus complaints for at least 6 months, ability to read and understand written Turkish, and complete the questionnaire form.

The exclusion criteria were as follows: diagnosed psychiatric illness (e.g., schizophrenia, bipolar disorder, major depression), neurological illness (epilepsy, Parkinson's disease, multiple sclerosis, etc.) reported by the participant, difficulty understanding the questionnaire items. All participants who were diagnosed with chronic subjective tinnitus were asked to complete the following questionnaires: the Tinnitus Handicap Inventory, the Tinnitus Acceptance Questionnaire, the Beck Depression Inventory, and the Pittsburgh Sleep Quality Index.

### 2.2 Data Collection Tools

#### 2.2.1 The Tinnitus Handicap Inventory (THI)

The Tinnitus Handicap Inventory (THI): The THI is a scale consisting of 25 questions, which is widely utilised in clinical settings. Each item is answered as follows: “yes” (4 points), “no” (0 points) or “sometimes” (2 points). The total score varies between 0 and 100. The higher the THI score, the greater the tinnitus‐related handicap. THI has subdomains, including emotional, functional, and catastrophic, and the tinnitus level is determined by the total THI score (Levels 1–5) (Newman et al. 1996). The Turkish version of the questionnaire demonstrated satisfactory internal consistency (Cronbach's alpha = 0.88) and test‐retest reliability (Aksoy et al. 2007).

#### 2.2.2 Tinnitus Acceptance Questionnaire (TAQ)

The TAQ is a 12‐item questionnaire designed to measure acceptance of tinnitus. Eight of the items are reverse‐scored, and the total score ranges from 0 to 72, with higher values indicating higher levels of acceptance. The Tinnitus Assessment Questionnaire has a two subscales, with the first subscale being “Activity engagement (AE)” and the second subscale being “Tinnitus suppression (TS)”. The first subscale, “Activity engagement,” is associated with behavioural activation and assesses the presence or degree to which a person can continue activities of daily living independent of tinnitus. The second subscale, “Tinnitus suppression,” measures attempts to control cognitions and emotions related to tinnitus and therefore represents a measure of experiential avoidance (Weise et al. 2013). The reliability assessment of the Turkish version revealed a Cronbach's alpha of 0.71 and confirmed the measure's reproducibility through test–retest analysis (Aydoğan et al. 2025).

#### 2.2.3 Beck Depression Inventory (BDI)

The BDI was developed by Beck in 1961 as a self‐report scale to measure the emotional, cognitive, somatic and motivational components of depression (Beck et al. 1961). It is one of the most frequently used self‐reporting tools in research and clinical settings, with a primary focus on the comprehensive evaluation of depression symptoms. The scale consists of 21 items, two of which are for emotions, 11 for cognitions, two for behaviours, five for physical symptoms and one for interpersonal symptoms. Patients are asked to choose the question that best reflects their situation. Each question is scored from 0 to 3, with scores ranging from 0 to 63. The results are evaluated as 0–9 as none/minimal depression, 10–18 as mild depression, 19–29 as moderate depression and 30–63 as severe depression (Sorias 1998). The Beck Depression Inventory (BDI), used to assess the severity of depressive symptoms, was validated and its reliability examined for the Turkish population by Teğin (Tegin 1987).

#### 2.2.4 Pittsburgh Sleep Quality Index (PSQI)

The PSQI was developed by Buysse and colleagues in 1989 (Buysse et al. 1989) and consists of 24 questions designed to evaluate subjective sleep quality. Sleep quality is comprised of seven subcomponents: habitual sleep efficiency, sleep latency, daytime dysfunction, sleep duration, sleep disorders, and use of sleeping pills. Each component is evaluated on a 0–3 scale. The total score of the seven components gives the total scale score, which varies between 0 and 21. The validity and reliability of the Pittsburgh Sleep Quality Index for the Turkish population were assessed by Agargün (1996).

### 2.3 Statistical Analysis

The data analysis of the study was evaluated in IBM SPSS v27 (IBM Corp., Armonk, New York, USA) statistical package program. If the data is continuous, it is expressed with mean, standard deviation, median, minimum and maximum, while categorical variables are expressed with frequency (*n*) and percentage (%). Pearson's correlation analysis was performed for the relationship between the questionnaire scores. Before conducting the regression analyses, the assumptions of normality, linearity, and independence were tested. The variables met the assumptions of normality and linearity, correlations confirmed linear relationships between THI, BDI, and PSQI scores, and no violations of multicollinearity were observed. Thus, the use of multiple and logistic regression analyses was considered appropriate. The predictive effect of THI, BDI and PSQI scores on TAQ score was examined using linear regression analysis. Results are presented as *B* regression coefficients, standard error of B (SE), 95% confidence intervals, Beta standardised regression coefficients, *t* statistics, and *p*‐values. A value of *p* < 0.05 was considered to be statistically significant. Additionally, logistic regression analysis was performed to examine the predictive effect of THI, PSQI, and BDI scores on tinnitus acceptance levels. The TAQ score was dichotomised into “low acceptance” and “high acceptance” groups based on the median value. The model's discriminative ability was evaluated using ROC analysis. Finally, the mediation analysis was conducted to test whether TAQ mediated the relationship between BDI and THI. It was hypothesised that depression acts as a psychological predictor, increasing tinnitus burden by affecting acceptance levels. PSQI was not included in the model as a predictor because it was considered a comorbid outcome variable rather than a cause of tinnitus. The average causal mediation effect (ACME), average direct effect (ADE), total effect, and proportion mediated were estimated using nonparametric bootstrapping with 5000 resamples to obtain bias‐corrected 95% confidence intervals.

## 3 Results

Descriptive statistics for questionnaire scores are presented in Table 1. A subsequent investigation of the mean THI score revealed that participants generally perceived the impact of tinnitus on quality of life to be moderate. Participants with PSQI scores above 5 indicate that sleep quality is generally poor. The data on depression levels indicate that the majority of the participants exhibited mild depressive symptoms. Furthermore, the level of acceptance of tinnitus was moderate among the participants.

**TABLE 1:** Descriptive statistics of questionnaire scores.

	Mean ± SD	Median (IQR)	Min–Max
**THI score**	48.69 ± 25.51	46	[0–100]
**PSQI score**	7.02 ± 3.42	7	[1–18]
**BDI score**	13.31 ± 9.1	11.5	[0–53]
**TS score**	8.81 ± 5.57	8	[0–38]
**AE score**	34.01 ± 10.81	35	[7–54]
**TAQ score**	42.3 ± 12.39	41	[6–69]

THI: Tinnitus Handicap Inventory; BDI: Beck Depression Inventory; PSQI: Pittsburgh Sleep Quality Index; TAQ: Tinnitus Acceptance Questionnaire; AE: activity engagement; TS: tinnitus suppression; SD: standard deviation; Min: minimum; Max: maximum; IQR: interquartile range.

The categorical distributions of THI, BDI, and PSQI levels are presented in Table 2. With regard to THI levels, the majority of participants were classified as Level 4 (severe), accounting for 26.2% of the sample, while the second most prevalent group was at Level 3 (moderate), constituting 25.4% of the total. Approximately 51.6% of the participants indicated that the impact of tinnitus on their quality of life was severe. With regard to BDI levels, the majority of participants were classified as “mild depression” (37.7%), followed by “minimal depression” (36.9%). This finding suggests that the majority of the participants exhibited mild depressive symptoms. The PSQI levels were analysed, revealing that 61.5% of the participants’ sleep quality was in the “bad sleep quality” category. This finding indicates that the majority of the participants experienced sleep problems.

**TABLE 2:** Descriptive statistics of THI, BDI, and PSQI levels.

	*n*	%
**THI level** Level 1 (very mild)	17	13.1
Level 2 (mild)	29	22.3
Level 3 (moderate)	33	25.4
Level 4 (severe)	34	26.2
Level 5 (catastrophic)	17	13.1
**BDI level** Minimal depression	48	36.9
Mild depression	49	37.7
Moderate depression	27	20.8
Severe depression	6	4.6
**PSQI level**		
Poor sleep quality	80	61.5
Good sleep quality	50	38.5

THI: Tinnitus Handicap Inventory; BDI: Beck Depression Inventory; PSQI: Pittsburgh Sleep Quality Index.

### 3.1 Correlation Analyses

The relationships between TAQ and other variables were examined using Pearson's correlation coefficients and are shown in Table 3.

**TABLE 3:** Pearson's correlation coefficients between questionnaire scores and levels (*n* = 130).

		TAQ score	AE score	TS score	THI score	BDI score	PSQI score
**TAQ score**	*r*	1	0.861^**^	0.297^**^	−0.667^**^	−0.438^**^	−0.401^**^
***AE score***	*r*		1	0.001	−0.667^**^	−0.461^**^	−0.380^**^
***TS score***	*r*			1	−0.091	−0.117	0.036
**THI score**	*r*				1	0.495^**^	0.439^**^
**BDI score**	*r*					1	0.516^**^
**PSQI score**	*r*						1
		**TAQ Score**	**AE Score**	**TS Score**	**THI Level**	**BDI Level**	**PSQI Level**
**TAQ score**	*r*	1	0.861^**^	0.297^**^	−0.632^**^	−0.445^**^	−0.351^**^
***AE Score***	*r*		1	0.001	−0.642^**^	−0.462^**^	−0.369^**^
***TS Score***	*r*			1	−0.083	−0.084	0.01
**THI level**	*r*				1	0.446^**^	0.395^**^
**BDI level**	*r*					1	0.337^**^
**PSQI level**	*r*						1

THI: Tinnitus Handicap Inventory; BDI: Beck Depression Inventory; PSQI: Pittsburgh Sleep Quality Index; TAQ: Tinnitus Acceptance Questionnaire; AE: activity engagement; TS: tinnitus suppression; *r*: Pearson's correlation coefficient.

* *p* < 0.05.

** *p* <0.01.

A subsequent examination of the correlation coefficients between the scales revealed significant strong negative correlations between the TAQ total score and the THI score, and moderate negative correlations with the BDI and PSQI scores (*p* < 0.05). The findings indicated that the TAQ total score was negatively correlated with the THI, BDI, and PSQI scores. The magnitude of the correlation between the TAQ total score and the THI score was notably strong (*r* = –0.667, *p* < 0.001), while the correlations with the BDI score (*r* = –0.438, *p* < 0.001) and the PSQI score (*r* = –0.401, *p* < 0.001) were moderate. These significant negative correlations indicate that higher tinnitus impact, depression, and sleep disturbance were associated with lower tinnitus acceptance. These findings are presented in Table 3.

When the correlation coefficients of the TAQ total score, AE score, and TS score with the THI, BDI, and PSQI levels were examined, significant negative correlations were observed with the THI, BDI, and PSQI levels (*p* < 0.05). The data revealed a strong negative relationship between the TAQ total score and the THI level (*r* = –0.632, *p* < 0.001), a moderate negative relationship with the BDI level (*r* = –0.445, *p* < 0.001), and a moderate negative relationship with the PSQI level (*r* = –0.351, *p* < 0.001), as shown in Table 3.

**FIGURE 1:**
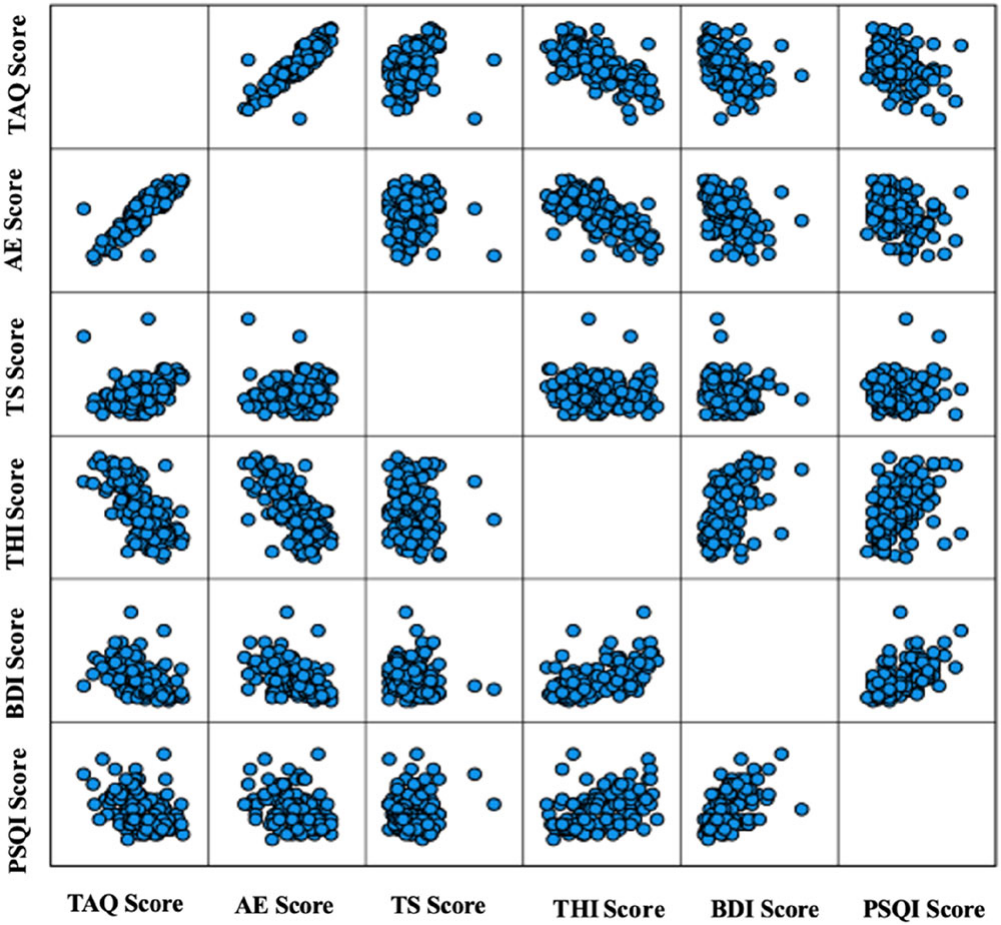
Correlation matrix plot of scores.

**FIGURE 2:**
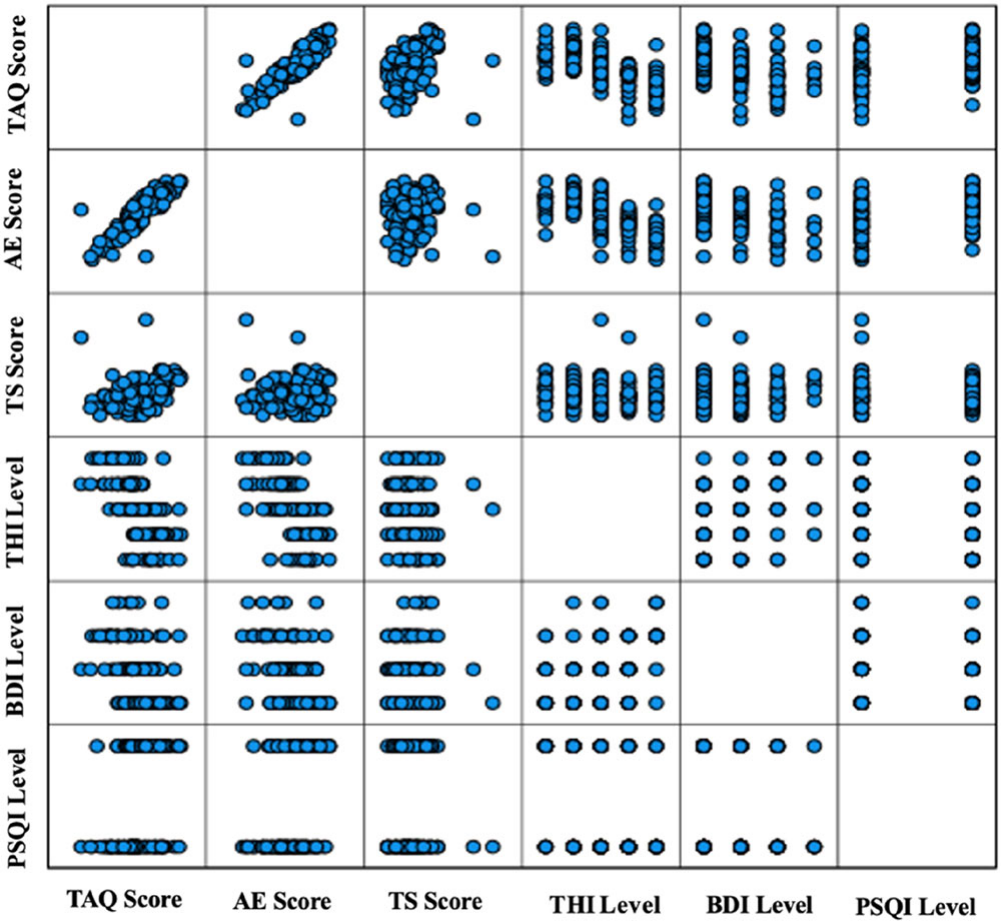
Correlation matrix plot of levels.

The correlation matrix graph of the scores (see Figure 1) and the correlation matrix graph of the levels (see Figure 2) are provided, illustrating the relationships between all questionnaire scores.

### 3.2 Regression Analyses

The results of multiple linear regression analyses examining the associations of THI, BDI, and PSQI on TAQ scores are shown in Table 4. The coefficient of regression *B* is indicative of the effect values. Among the predictors, THI was found to have a significant negative effect on the dependent variable (*B* = –0.28, SE = 0.04, *t* = –7.42, *p* < 0.001, 95% CI [–0.35, –0.20]). This indicates that for each one‐unit increase in THI score, the dependent variable decreases by approximately 0.28 units, holding other variables constant.

**TABLE 4:** Multiple linear regression analysis of the effect of THI, BDI, and PSQI scores on TAQ score.

Predictor	*B* (estimate)	SE	*t*	*p*‐value	95% CI lower	95% CI upper
**(Intercept)**	60.17	2.05	29.35	< 0.001	56.11	64.23
**THI**	−0.28	0.04	−7.42	**< 0.001**	−0.35	−0.20
**BDI**	−0.14	0.11	−1.29	0.198	−0.36	0.08
**PSQI**	−0.34	0.28	−1.21	0.228	−0.90	0.22

Model summary: residual SE = 9.16; *R* ^2^ = 0.47; adjusted *R* ^2^ = 0.45; *F*(3, 126) = 36.69; *p* < 0.001.

THI: Tinnitus Handicap Inventory; BDI: Beck Depression Inventory; PSQI: Pittsburgh Sleep Quality Index; TAQ: Tinnitus Acceptance Questionnaire; *B* = unstandardised regression coefficient; SE: standard error; *t*: *t*‐value.

Bold values indicate statistically significant results (*p* <0,005).

In contrast, BDI (*B* = –0.14, SE = 0.11, *t* = –1.29, *p* = 0.198, 95% CI [–0.36, 0.08]) and PSQI (*B* = –0.34, SE = 0.28, *t* = –1.21, *p* = 0.228, 95% CI [–0.90, 0.22]) were not significant predictors of the dependent variable.

### 3.3 Logistic Regression Analysis

Since the tinnitus acceptance score is a continuous variable, it was divided into “high” and “low” groups according to the median value, in order to meet the requirement for a binary dependent variable in the regression analysis. When there is no clinically determined threshold value, the median division method is a practical classification method that is frequently preferred, especially in behavioural sciences and health research (MacCallum et al. 2002; Streiner 2002).

The median TAQ score was found to be 41.0. In this case, TAQ scores of ≤41 were categorised as “low acceptance,” while scores of >41 were categorised as “high acceptance.” After dividing the tinnitus acceptance level into low and high groups according to the median, logistic regression analysis showed that the model was statistically significant (*p* < 0.001). The THI score was found to be a significant predictor of tinnitus acceptance level (*β* = –0.047, *p* < 0.001). Each unit increase in the THI score decreased the probability of having a high tinnitus acceptance level by approximately 5% (odds ratio [OR] = 0.95; 95% confidence interval [CI]: 0.93–0.98). However, the contribution of the PSQI and BDI scores to the model was not found to be statistically significant (*p* > 0.05) (see Table 5). After that, the discriminative performance of the logistic regression model was evaluated using ROC and classification metrics. The model demonstrated a good level of discrimination, with an overall accuracy of 0.84 (95% CI [0.76–0.90]) (see Table 6)

**TABLE 5:** Logistic regression results for predicting tinnitus acceptance levels based on THI, PSQI, and BDI scores.

Variable	*B* (*β*)	SE	*p*	OR	95% CI (lower)	95% CI (upper)
Intercept	3.8242	0.7150	0.0000	45.7976	11.2783	185.9695
THI	−0.0470	0.0111	**0.0000**	0.9541	0.9336	0.9751
PSQI	−0.0828	0.0766	0.2798	0.9205	0.7922	1.0697
BDI	−0.0533	0.0326	0.1015	0.9481	0.8895	1.0105

THI: Tinnitus Handicap Inventory; BDI: Beck Depression Inventory; PSQI: Pittsburgh Sleep Quality Index; *B* = regression coefficient (beta); SE: standard error; OR: odds ratio; CI: confidence interval.

Bold values indicate statistically significant results (*p* <0,005).

**TABLE 6:** Classification performance metrics of the logistic regression model.

Metric	Value
Accuracy	0.838
95% CI	0.763–0.897
Kappa	0.671
Sensitivity	0.901
Specificity	0.763
Positive predictive value (precision)	0.821
Negative predictive value	0.866
*F*1 score	0.86
Balanced accuracy	0.832

### 3.4 Mediation Analysis

**FIGURE 3:**
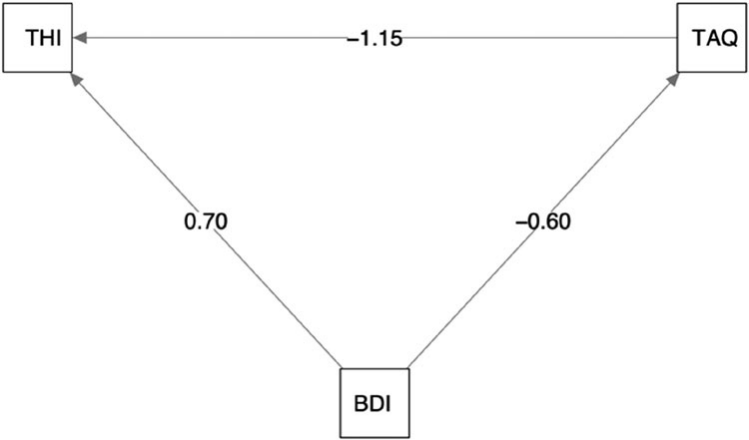
Path diagram of the mediation model.

The indirect effect of depression on THI through TAQ was statistically significant (ACME = 0.68, 95% CI [0.39, 1.09], *p* < 0.001). The direct effect of BDI on THI, after controlling for acceptance, remained significant (ADE = 0.70, 95% CI [0.25, 1.19], *p* = 0.004). The total effect of BDI on THI was also significant (total effect = 1.39, 95% CI [0.91, 1.92], *p* < 0.001). Approximately 49% of the total effect of BDI on THI was transmitted through TAQ (proportion mediated = 0.49, 95% CI [0.30, 0.78], *p* < 0.001) (see Table 7 and Figure 3).

**TABLE 7:** Mediation analysis results.

Effect type	Estimate	95% CI lower–upper	*p*‐value
**ACME (indirect effect)**	0.68	0.39–1.09	**< 0.001**
**ADE (direct effect)**	0.70	0.25–1.19	0.0036
**Total effect**	1.39	0.91–1.92	**< 0.001**
**Proportion mediated**	0.49	0.30–0.78	**< 0.001**

ACME: average causal mediation effect, ADE: average direct effect.

Bold values indicate statistically significant results (*p* <0,005).

Mediation analysis indicated that TAQ played a partial mediating role in the relationship between depression (BDI) and tinnitus disability (THI). As depression levels increased, acceptance levels decreased, and this decrease in acceptance was associated with a higher tinnitus burden. Because both the indirect effect (ACME = 0.68, *p* < 0.001) and the direct effect (ADE = 0.70, *p* = 0.004) were significant, the model supports partial mediation.

Path diagram Mediation model illustrating the proposed mediation model. Rectangles represent observed variables: BDI (Beck Depression Inventory), TAQ (Tinnitus Acceptance Questionnaire), and THI (Tinnitus Handicap Inventory). Single‐headed arrows indicate hypothesised directional effects between variables. The arrow from BDI to TAQ represents the effect of depression on tinnitus acceptance, the arrow from TAQ to THI represents the effect of acceptance on tinnitus handicap, and the direct arrow from BDI to THI represents the direct pathway between depression and tinnitus handicap. Numerical values on the paths denote unstandardised regression coefficients obtained from the mediation analysis.

### 3.5 Additional Analyses

Additional analyses were performed to examine the potential conceptual overlap between TAQ and THI. The two measures showed a strong negative correlation (*r* = –0.67, *p* < 0.001, 95% CI [–0.75, –0.56]). However, variance inflation factors (VIF) from a multiple regression model including THI, PSQI, BDI, age, and gender were all below 2 (VIF for THI = 1.42), indicating no multicollinearity concerns. These findings suggest that TAQ and THI, while conceptually related, assess distinct psychological dimensions of tinnitus (acceptance vs. perceived impact).

## 4 Discussion

In this study, the association between tinnitus acceptance, sleep quality and depression were examined, and the results obtained showed a significant association between the level of acceptance and the quality of life of individuals. Significant relationships were identified between variables such as tinnitus severity, depression level, sleep quality and acceptance. It was determined that the degree of tinnitus‐ related distress experienced by the individual was significantly associated with the acceptance process. Regression analyses identified THI as the strongest predictor of tinnitus acceptance, while BDI and PSQI were not independent predictors. The ROC analysis further supported the discriminative ability of acceptance scores in differentiating between low and high tinnitus‐related distress levels. In addition, the mediation analysis showed that depression partially mediated the association between tinnitus acceptance and tinnitus handicap, whereas sleep quality did not. The subsequent discussion addressed the consistency of these findings with previous literature, potential clinical applications, and the benefits of acceptance‐based interventions.

The adaptation process to phantom perception caused by tinnitus varies from individual to individual, but in some patients this situation can lead to a serious decrease in quality of life and additional problems such as anxiety, stress, depression or sleep disorders (Møller 2007). Research has highlighted that tinnitus perception is predominantly influenced by psychological factors, including thoughts, emotions, personality traits and coping strategies (Durai and Searchfield 2016; Ooms et al. 2012). Tinnitus acceptance, measured by TAQ, has been identified as a fundamental construct in tinnitus research. It has been proposed that this construct should be further integrated into treatment approaches with a view to alleviating tinnitus‐related distress (Weise et al. 2013).

The descriptive statistics and categorical distributions show that participants generally perceive the impact of tinnitus on their quality of life to be moderate. The assessment of tinnitus impact shows that participants perceive its effect on their quality of life to be significant. Sleep quality scores show that most participants have poor sleep quality compared with the average score of the Pittsburgh Sleep Quality Index normative sample, suggesting a possible relationship between tinnitus and poorer sleep patterns. Depressive symptom scores show that most of the participants have mild depressive symptoms. In addition, the moderate level of tinnitus acceptance shows that participants have difficulties in coping with tinnitus, but have partially accepted this situation. The impact of tinnitus on quality of life and the relationship between depression and sleep quality may provide an important basis for psychosocial interventions.

In the present study, THI was the strongest predictor of tinnitus acceptance level, indicating that individuals with higher tinnitus‐ related handicap tended to report lower acceptance. This finding suggests that greater tinnitus severity is associated with reduced acceptance levels. Many studies have shown the relationship between tinnitus disability level and depression (Molnár et al. 2022). Research findings indicate that individuals with tinnitus exhibit significantly higher levels of anxiety and depression compared to the general population (Pattyn et al. 2016). In our study, 36.9% of individuals with tinnitus had minimal depression, 37.7% had mild depression, 20.8% had moderate depression, and 4.6% had severe depression. Although the regression analysis conducted in our study did not identify depression (BDI score) as a significant predictor of tinnitus acceptance, correlation analysis revealed a moderate negative relationship between the two. In contrast, mediation analysis indicated that depression played a significant indirect role in the association between tinnitus acceptance and tinnitus handicap, suggesting that depressive symptoms may partially mediate this relationship. A study consistent with this findings demonstrated that acceptance exhibited a strong inversely proportional correlation with tinnitus severity, anxiety, and depression symptoms (Hesser et al. 2015). The provision of psychological support and/or depression management has been shown to facilitate the acceptance process (Moschen et al. 2015). The emerging data emphasise the positive effects of adding acceptance‐based approaches to behavioural therapies in health problems that create chronic stress (Vendela Zetterqvist Westin et al. 2011). It is reported in the literature that cognitive behavioural therapies are effective in reducing tinnitus awareness and alleviating the negative effects of this condition on individuals' quality of life (Cima et al. 2012). Concurrently, the results of the present study demonstrated a positive correlation between the severity of depression and the quality of sleep. A positive association was observed between the severity of depression and sleep quality, indicating that as depression severity increases, sleep quality tends to deteriorate; however, this relationship may also operate in the opposite direction or be influenced by other factors. Research findings suggest that sleep disturbances can both contribute to the development of anxiety and enhance sensitivity to emotional challenges in individuals suffering from tinnitus (Richter et al. 2021). Interventions designed to enhance acceptance therefore have the potential to alleviate depressive symptoms and improve sleep quality.

Sleep plays an important role in the physiological and psychological balance of the individual (Monroe 1967). According to studies in the literature, the prevalence of sleep problems in individuals with tinnitus varies between 37% and 73% (Inagaki et al. 2021; Xu et al. 2016). It the present study, 61.5% of the participants were found to have poor sleep quality. Prior studies have indicated a positive correlation between tinnitus severity and disrupted sleep (Asplund 2003; Miguel et al. 2014). However, the relationship between tinnitus acceptance and sleep quality remains to be elucidated. Similarly, our findings revealed a moderate negative correlation between sleep quality and tinnitus acceptance, indicating that individuals with poorer sleep quality tended to report lower acceptance levels. However, this relationship was not significant in regression or logistic regression analyses, suggesting that sleep quality may not independently predict tinnitus acceptance when other factors, such as tinnitus severity and depression, are considered. Moreover, sleep quality (PSQI) was not included in the mediation model, as it was conceptualised as a comorbid outcome variable rather than a causal predictor of tinnitus acceptance. It has been established that sleep disorders have a deleterious impact on stress tolerance and coping mechanisms (Han et al. 2012). A randomised controlled study reported that Acceptance and Commitment Therapy significantly reduced stress, sleep problems, and anxiety caused by tinnitus, and that these improvements were largely achieved through “tinnitus acceptance” (V. Z. Westin et al. 2011).

Research has demonstrated that the assessment of acceptance processes among individuals with tinnitus constitutes a valuable metric in experimental investigations of psychological treatments for tinnitus. Tinnitus‐related acceptance has been associated with enhanced functioning and well‐being (Hesser et al. 2015; Westin et al. 2008). A study by Schutte et al. (2009) found a significant relationship between tinnitus acceptance and lower levels of tinnitus‐related distress (Schutte et al. 2009). In the present study, THI, BDI, and PSQI scores were found to be significant correlations with TAQ scores. THI was found to be the strongest factor associated with acceptance, highlighting the substantial influence of tinnitus burden on an individual's quality of life. The negative correlations observed with BDI and PSQI indicate that higher depression and poor sleep quality are related to lower acceptance. The correlations identified between the tinnitus‐specific acceptance measure and depression and sleep problems reflect how these psychological factors relate to perceptions of tinnitus discomfort. The findings are consistent with existing literature and highlight the association between tinnitus acceptance and better psychological and physical health indicators. Consequently, psychological support or treatment approaches that address depression and sleep quality may contribute to improving the tinnitus acceptance process. Furthermore, given that tinnitus acceptance is strongly associated with reduced distress and better psychological functioning (Hesser et al. 2015; Schutte et al. 2009) and that our findings indicate a significant relationship between acceptance, depression, and sleep quality, the development of therapeutic interventions specifically targeting the impact of tinnitus on quality of life represents a promising research direction. Evidence from cognitive‐behavioural and acceptance‐based interventions suggests that enhancing acceptance may help improve coping and overall well‐being in tinnitus patients (Cima et al. 2012; Westin et al. 2008). A key tenet of cognitive‐behavioural therapy is acceptance, which encourages individuals to embrace tinnitus as a part of their life rather than attempting to combat it. This approach has the potential to enhance emotion regulation processes and alleviate depressive symptoms.

It is important to note that there is a considerable conceptual overlap between some of the measurement instruments employed in this study. For instance, several instruments encompass items pertaining to sleep quality, and both the TAQ and THI address analogous aspects of tinnitus impact and coping, albeit in slightly divergent ways. This overlap may have contributed, at least in part, to the observed associations between these measures, as correlations could reflect shared content rather than entirely distinct constructs. To further examine this issue, supplementary analyses were conducted to control for potential overlap. Partial correlation and multicollinearity tests indicated that the associations among the main variables remained significant even after accounting for shared variance, implying that the relationships observed are not solely attributable to overlapping item content. It is recommended that future research address this issue by repeating the analyses with overlapping items removed which would allow for a clearer examination of whether the relationships persist when redundancy is minimised.

The present study is subject to several limitations. The most significant limitation is its cross‐sectional design, which precludes any conclusions about causal relationships or the temporal sequence between tinnitus acceptance, depression, and sleep quality. Due to the fact that the data were collected at a single point in time, it is not possible to determine whether changes in one variable precede or result from changes in another. Furthermore, a substantial conceptual overlap exists among certain measurement instruments. For instance, several instruments encompass items pertaining to sleep quality, and both the TAQ and THI address analogous aspects of tinnitus impact and coping, albeit in slightly divergent ways. This overlap may have contributed, at least in part, to the observed associations between these measures. Furthermore, the utilisation of self‐report scales may have engendered individual perception differences in the responses of the participants, and the generalisability of the findings may be constrained by the demographic and cultural characteristics of the study participants.

## 5 Conclusion

The present findings highlight potential links between tinnitus acceptance, depression, and sleep quality. These relationships are correlational and should be interpreted with caution. Integrating acceptance‐based elements into psychological support programs may be a promising approach, which warrants further confirmation in longitudinal and interventional studies.

## Author Contributions

Conceptualization: Sevgi Kutlu, Zehra Aydogan Methodology:Sevgi Kutlu, Zehra Aydogan, Kübra Binay Bolat, Nazife Öztürk Özdes Investigation: Sevgi Kutlu, Zehra Aydogan, Kübra Binay Bolat, Nazife Öztürk Özdes Data Curation: Sevgi Kutlu, Zehra Aydogan, Kübra Binay Bolat, Nazife Öztürk Özdes Writing: Sevgi Kutlu Writing – Review and Editing: Sevgi Kutlu Visualization: Sevgi Kutlu Supervision: Sevgi Kutlu.

## Funding

The authors have nothing to report.

## Conflicts of Interest

The authors declare that there is no conflict of interest regarding the publication of this paper.

## Ethics Statement

The present study was conducted in accordance with the principles of the Declaration of Helsinki. The ethical approval of the study was obtained by the Ankara University Faculty of Medicine Human Research Ethics Committee (decision number: İ02‐108‐25).

## Patient Consent Statement

Informed consent was obtained from all individual participants included in the study.

## Permission to Reproduce Material from Other Sources

No material from other sources requiring permission was used in this manuscript.

## Clinical Trial Registration

This study is not a clinical trial and therefore was not registered.

## Ethical Compliance and Integrity Statement

The authors affirm that this manuscript adheres to the ethical guidelines of the journal and complies with accepted standards for research integrity, including the avoidance of plagiarism, data fabrication, and inappropriate authorship practices. All authors have reviewed and approved the final manuscript and take full responsibility for its content.

## Data Availability

The data that support the findings of this study are not publicly available due to ethical restrictions but are available from the corresponding author upon reasonable request.
